# Beyond Transconductance: Cell‐Polymer Coupling Determines Fidelity in Action Potential Recording via Electrolyte‐Gated Polymer Transistors

**DOI:** 10.1002/advs.202520122

**Published:** 2026-02-12

**Authors:** Giulia Zoe Zemignani, Elena Mancinelli, Gabriele Tullii, Aleksandr Khudiakov, Cristiano Bortolotti, Shubham Tanwar, Peter J. Schwartz, Luca Sala, Maria Rosa Antognazza, Mario Caironi, Adrica Kyndiah

**Affiliations:** ^1^ Center For Nano Science and Technology Istituto Italiano di Tecnologia Milano Italy; ^2^ Center For Cardiac Arrhythmias of Genetic Origin and Laboratory of Cardiovascular Genetics Istituto Auxologico Italiano IRCCS Milan Italy; ^3^ Department of Electronics Information and Bioengineering Politecnico di Milano Milano Italy; ^4^ Department of Biotechnology and Biosciences University of Milano‐Bicocca Milan Italy

**Keywords:** cardiomyocytes action potentials, electrophysiology, organic bioelectronics, printed polymer transistor

## Abstract

Organic bioelectronic transistors emerged as powerful tools for probing cellular electrophysiology, offering biocompatibility, mechanical softness, and operational stability in biological environments. Despite these advantages, device benchmarking focused so far exclusively on electrical figures of merit, leaving interfacial processes that contribute to signal recording fidelity largely unexplored. Poly[2‐(3,3′‐bis(2‐(2‐(2‐methoxyethoxy)ethoxy)ethoxy)‐[2,2’‐bithiophen]‐5‐yl)thieno[3,2‐b]thiophene] (p(g2T‐TT)) is a model glycolated organic mixed ionic‐electronic conductor, whose high transconductance and optimal volumetric field‐effect behavior in physiological environment have been widely documented in Organic Electrochemical Transistor (OECT) configurations. Thus, p(g2T‐TT)‐based OECT was assessed as a promising candidate for recording action potentials (APs) from human induced pluripotent stem cell‐derived cardiomyocytes (hiPSC‐CMs). Surprisingly, although AP signal transduction was observed, the recorded AP waveforms failed to reproduce the expected morphology, especially when compared to signals recorded via poly(3‐hexylthiophene‐2,5‐diyl) (P3HT)‐based Electrolyte‐Gated Field‐Effect Transistors (EGOFETs). Immunofluorescence imaging revealed improved adhesion on P3HT with respect to p(g2T‐TT), suggesting weaker cell‐device coupling as the underlying limitation. Our results show that not all polymers combining biocompatibility and high electrical performance can transduce AP signals with high‐fidelity. Instead, interfacial properties govern bioelectronic transduction and provide a foundation for the rational design of polymers and platforms enabling reliable in vitro cellular electrophysiology, with potential translation to in vivo applications.

## Graphical abstract

High electrical performance alone does not guarantee efficient bioelectronic signal transduction. Despite excellent mixed ionic‐electronic conduction, recordings from cardiomyocytes reveal that p(g2T‐TT)‐based OECTs predominantly transduce field potential FP‐like signals due to weak cell‐polymer coupling. In contrast, P3HT EGOFETs yield high‐fidelity action potential AP‐like signals, highlighting interfacial properties as a critical design parameter for organic bioelectronic interfacing.

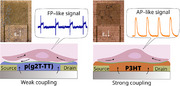

## 1 Introduction

Accurate, label‐free, and non‐invasive recording of action potentials (APs) from excitable cells, such as neurons, cardiomyocytes, and skeletal muscle cells, remains a central challenge in bioelectronics. The AP waveform, duration, and frequency serve as key physiological markers of a cell state. High‐fidelity monitoring of cellular electrophysiology in vitro is critical for fundamental research, disease modelling, and preclinical drug screening [1]. In this context, the development of bioelectronic platforms capable of transducing APs with high spatiotemporal resolution, biocompatibility, and long‐term stability is of paramount importance [2]. Among bioelectronic platforms, Electrolyte‐Gated Organic Transistors (EGOTs), including Electrolyte‐Gated Organic Field‐Effect Transistors (EGOFETs) and Organic Electrochemical Transistors (OECTs) based on conjugated polymers, have emerged as powerful tools for electrophysiology [3, 4]. Their favorable mechanical properties, long‐term biocompatibility, and stability in biological environments allow polymer‐based devices to overcome many of the limitations associated with silicon‐based technologies [5]. The key distinction between EGOFETs and OECTs lies in the role of ion permeation through the organic semiconductor. While in EGOFETs, ion penetration is negligible, and current modulation arises from the formation of an electric double layer at the electrolyte/semiconductor interface, in OECTs, instead, extensive ion penetration into the polymer bulk leads to the establishment of a volumetric capacitance, which governs the modulation mechanism [6, 7]. In both cases, however, electrolyte gating allows ion fluxes from biological events, such as APs, to modulate channel conductivity, directly converting them into electronic signals [8, 9, 10, 11, 12, 13]. In such platforms, cells are cultured directly on the semiconducting polymer surface and form a cleft whose lateral electrical resistivity strongly influences the fidelity of AP transduction [9, 13, 14]. Thus, reliable electrophysiological recording depends not only on the intrinsic electrical properties of the device, but also, equally important, on the quality of cell‐device coupling. Yet, benchmarking EGOTs for bioelectronic applications has largely centered on figures of merit such as transconductance, mobility, and capacitance, while giving comparatively little attention to the interfacial factors that ultimately govern signal fidelity [3, 7]. These factors, including (but not limited to) surface chemistry, wettability, morphology, and protein adsorption, critically influence cell adhesion to the substrate and, consequently, the efficiency with which biological signals are transferred to the device [15]. As a result, limitations at the biointerface may sizably affect recording performance. In fact, the plasma membrane actively mediates all the processes occurring at the interface between the cell and the extracellular matrix, enabling the response of the cell to environmental stimuli: recent studies indicate its central role in governing the overall efficiency of bioelectronic devices for both stimulation and sensing. Planar substrates, especially those based on conjugated polymers, can be tailored to achieve a tight engagement with the plasma membrane and a significant improvement of the cell‐chip coupling by adopting several strategies, as recently reviewed by F. Santoro et al., [16]. Those include roughness modulation, surface topography, wettability, and chemical functionalization with adhesive extracellular matrix proteins, either adsorbed, covalently linked, or even embedded within the polymer. It has been demonstrated that improved coupling between the cell and films of the conductive polymer, such as poly (3,4‐ethylenedioxythiophene) polystyrene sulfonate (PEDOT: PSS), has a significant impact on electrical conduction at the interface, allowing for higher fidelity electrophysiological recordings [17].

Despite the potential of these approaches, however, experimental results obtained in cell activity recording using planar architectures (either EGOFET or OECT) supported the widely accepted paradigm that to achieve intracellular‐like recording, it is necessary to physically penetrate the cell membrane [18]. This prompted huge technological efforts to realize minimally invasive, 3D nanoelectrodes, like pillars, nanoholes, grooves, and vertical rods (for a recent review of the tools specifically designed for cardiac electrophysiology, see ref. [19]).

In one of our recent works, we demonstrated that printed poly(3‐hexylthiophene‐2,5‐diyl) (P3HT)‐based EGOFETs can reliably transduce AP signals in human induced pluripotent stem cell‐derived cardiomyocytes (hiPSC‐CMs) in a non‐invasive manner, with patch clamp‐like fidelity, despite their planar geometry [14]. Available models of the cell‐transistor interface, including the point‐contact equivalent circuit coupled to the Luo‐Rudy ventricular cell model [20] used in our recent work, indicate that both the transistor transconductance and the cleft resistance critically determine the shape and signal‐to‐noise ratio (SNR) of AP recordings [14]. Focusing on transconductance, we investigated the use of poly[2‐(3,3′‐bis(2‐(2‐(2‐methoxyethoxy)ethoxy)ethoxy)‐[2,2’‐bithiophen]‐5‐yl)thieno[3,2‐b]thiophene] (p(g2T‐TT)) in OECTs for AP recordings, as this model glycolated organic mixed ionic‐electronic conductor (OMIEC), besides exhibiting an exceptionally high ionic mobility, shows, when used as active material in OECTs, transconductance values more than one order of magnitude higher than P3HT, thanks to its large volumetric capacitance [21, 22]. We found that, although p(g2T‐TT)‐based OECTs could transduce AP signals, the recorded waveforms failed to reproduce the typical AP morphology, in contrast to the accurate signals obtained with P3HT‐based EGOFETs. Such evidence reveals that intrinsic electrical figures of merit are insufficient, per se, to ensure high‐fidelity AP recordings. Instead, immunofluorescence analysis of vinculin localization, a protein involved in cell‐substrate interaction, revealed significantly better adhesion of hiPSC‐CMs on P3HT as compared to p(g2T‐TT), strongly suggesting cell‐device interfacial coupling as a major mechanism in AP signal recording. While a direct correlation between cell adhesion, cleft resistance, and signal transduction cannot be established in a straightforward manner, our findings highlight the central role of interface engineering in organic bioelectronics, beyond the mere optimization of intrinsic device electrical properties such as ionic mobility and transconductance. This insight opens the way to the rational design of next‐generation bioelectronic interfaces. Such advances could improve the reliability, fidelity, and longevity of in vitro electrophysiological platforms for drug screening, toxicity testing, and disease modeling, and extend high‐quality bioelectronic recordings to tissue‐level and in vivo applications.

## 2 Results and Discussion

### 2.1 p(g2T‐TT)‐based OECTs for AP Recordings of Cardiomyocytes

**FIGURE 1:**
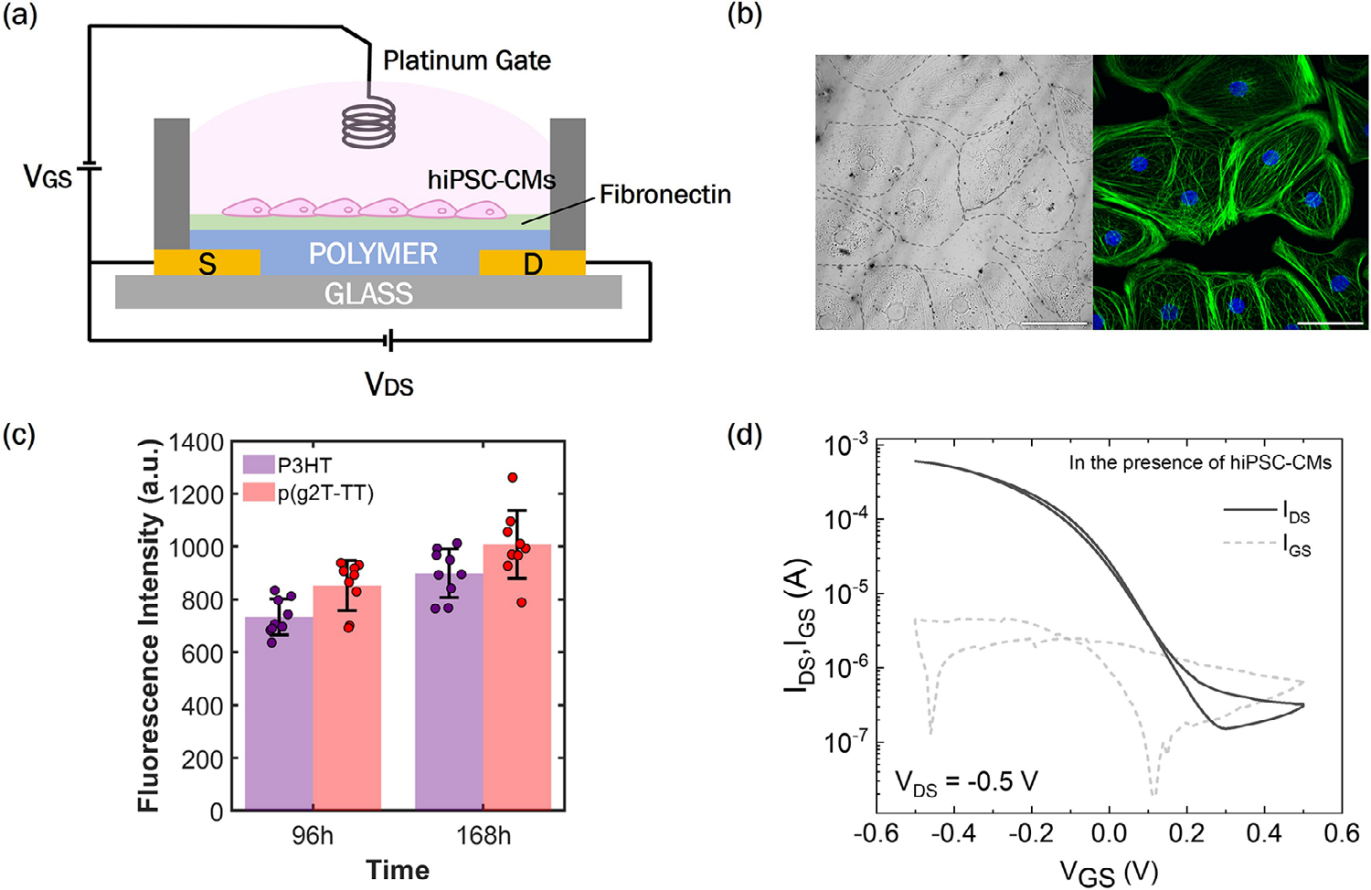
(a) Schematic representation of the lateral view of the device structure in the presence of a hiPSC‐CMs layer, where a gate electrode made of Pt is immersed into cell medium, which acts as the electrolyte. (b) Bright field (left) and fluorescent actin filament staining (right) of hiPSC‐CMs cultured on p(g2T‐TT) thin films. Outline of the cell perimeters is overlaid on the bright‐field image to guide the identification of cell boundaries. The nuclei and cytoskeleton (actin filaments) of the cells are stained with Hoechst (blue) and phalloidin‐FITC (green), respectively. Scale bars: 50 µm. (c) Viability of hiPSC‐CMs was evaluated as the fluorescence of the reduced form of the AlamarBlue cell‐viability reagent. Error bars represent the standard deviation (N = 9, three experimental replicas per substrate). (d) Representative transfer characteristic of p(g2T‐TT)‐based OECTs in the presence of hiPSC‐CMs. The gate voltage is swept from +0.5 to −0.5 V, and V_DS_ is set to −0.5 V. The transfer characteristic was acquired using a gate‐voltage sweep rate of approximately 1.25 mV/ms, using RBK1 cell culture medium as the electrolyte (see Experimental Section for composition).

A schematic representation of the OECT architecture employed for AP recording is depicted in Figure 1a. It consists of interdigitated source‐drain electrodes made of gold, fabricated by photolithography on glass to obtain a channel width (*W*) of ∼ 2 cm and channel length (*L*) of 10 µm (see Figure SI.1 for detailed description). A thin film of p(g2T‐TT) was then deposited on top of the electrodes by means of ink‐jet printing. To promote hiPSC‐CM adhesion, the polymer was coated with fibronectin prior to cell plating. The hiPSC‐CMs were subsequently plated on the device and kept in cell culture maintenance medium. A platinum coil served as the gate electrode, completing the OECT configuration. The OECT geometry was the same as that of previously reported EGOFETs based on P3HT [14]. Before performing electrical measurements, the presence of hiPSC‐CMs was assessed using immunofluorescence imaging. Cardiomyocytes plated on p(g2T‐TT) display the typical morphology of hiPSC‐CMs [23], as shown by the representative bright field and fluorescence images reported in Figure 1b. Long‐term viability of hiPSC‐CMs cultured on p(g2T‐TT) was then evaluated by AlamarBlue and live/dead assays. The AlamarBlue compound is based on resazurin, a non‐fluorescent reagent that permeates living cells and, once internalized, is reduced to the highly fluorescent resorufin. The fluorescence of the latter is directly proportional to the viability and proliferation of the cells. Cellular viability was evaluated 96 and 168 h after plating. For comparison, the same experiment was repeated with P3HT, successfully adopted to record APs of hiPSC‐CMs in our previous work, and acting here as a control sample given its high in vitro and in vivo biocompatibility [24, 25, 26, 27, 28, 29]. As illustrated in Figure 1c, p(g2T‐TT) and P3HT samples display a comparable AlamarBlue fluorescence, both after 96 and 168 h, highlighting the absence of detrimental effects on hiPSC‐CMs survival rate. To further prove the compatibility of p(g2T‐TT) with hiPSC‐CM cultures, we performed a live/dead fluorescence assay 168 h after plating, and we obtained 98% of viable cells on p(g2T‐TT) substrate (Figure SI.2).

We then moved to electrical measurements. A typical transfer characteristic curve of p(g2T‐TT) devices in the presence of hiPSC‐CMs is reported in Figure 1d. The optimized ink formulation and printing parameters used here were previously shown to yield uniform films and state‐of‐the‐art device performance [22, 30]. The *I–V* transfer characteristics, measured at drain‐source voltage *V*_DS_ = −0.5 V, display minimal hysteresis between forward and reverse gate‐source voltage *V*_GS_ sweeps (black trace, left axis). Across the measured p(g2T‐TT) OECTs, the maximum transconductance *g_m_,_max_*, extracted from N = 7 devices, is  1.7 ± 0.6 mS (mean value ± standard deviation).

**FIGURE 2:**
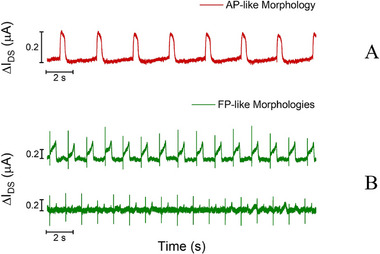
Representative biological signals recorded using p(g2T‐TT)‐based OECTs. *V_DS_* is set to −0.5 V while *V_GS_* is in the range of the maximum transconductance. Type A represents an AP‐like morphology, while type B displays two representative FP‐like morphologies obtained from the electrical recordings.

To record APs with p(g2T‐TT)‐based OECTs, we monitored the drain‐source current over time (*I_DS_* (*t*)), keeping the source electrode grounded, *V_DS_* =  − 0.5 V, and *V_GS_* close to the peak of *g*_*m*_. Representative Δ*I_DS_* (*t*) traces acquired from p(g2T‐TT) devices are shown in Figure 2. Each trace displays a periodic modulation of the current whose frequency corresponds to the spontaneous rhythmic activity of hiPSC‐CMs. However, the recorded signals do not share the same morphology and can be broadly categorized into two groups based on their waveform: type A corresponding to the AP‐like morphology (Figure 2a) and type B corresponding to Field Potential (FP)‐like morphologies (Figure 2b). The AP‐like signals obtained with p(g2T‐TT)‐based OECTs were rare. ∼15% of the overall recorded signals start with an AP‐like morphology, but degrade fast over time and evolve into FP‐like shapes, unlike the stable AP signals obtained using P3HT‐based EGOFETs (Figure SI.3). Only ∼8% of the total signals show a persistent AP‐like waveform for the time of measurement considered. FP‐like signals (type B), instead, exhibited variability in their form depending on the cell‐polymer coupling, as previously reported in Microelectrode Arrays (MEAs) recordings on hiPSC‐CMs [31]. While p(g2T‐TT)‐based OECTs demonstrated the ability to capture electrophysiological signals from cells, most of the recorded waveforms (∼77%) showed FP‐like characteristics. These waveforms displayed significant morphological variations and inconsistent SNR, suggesting notable variability, even within a single array of transistors. Examples of the FP‐like heterogeneity observed are reported in Figure SI.4. This result contrasts with our earlier work with P3HT‐based EGOFETs, where over 90% of recorded signals represented APs [14]. A quantitative analysis was performed to compare AP‐like vs FP‐like morphologies (Figure SI.5). AP‐like signals enabled the extraction of a broader set of temporal parameters, such as the AP durations (APD) at different phases of the repolarization. These parameters are critical for in vitro disease modelling and drug testing and cannot be derived from FP‐like signals.

### 2.2 Comparative AP Recordings with p(g2T‐TT) and P3HT Active Layers

**FIGURE 3:**
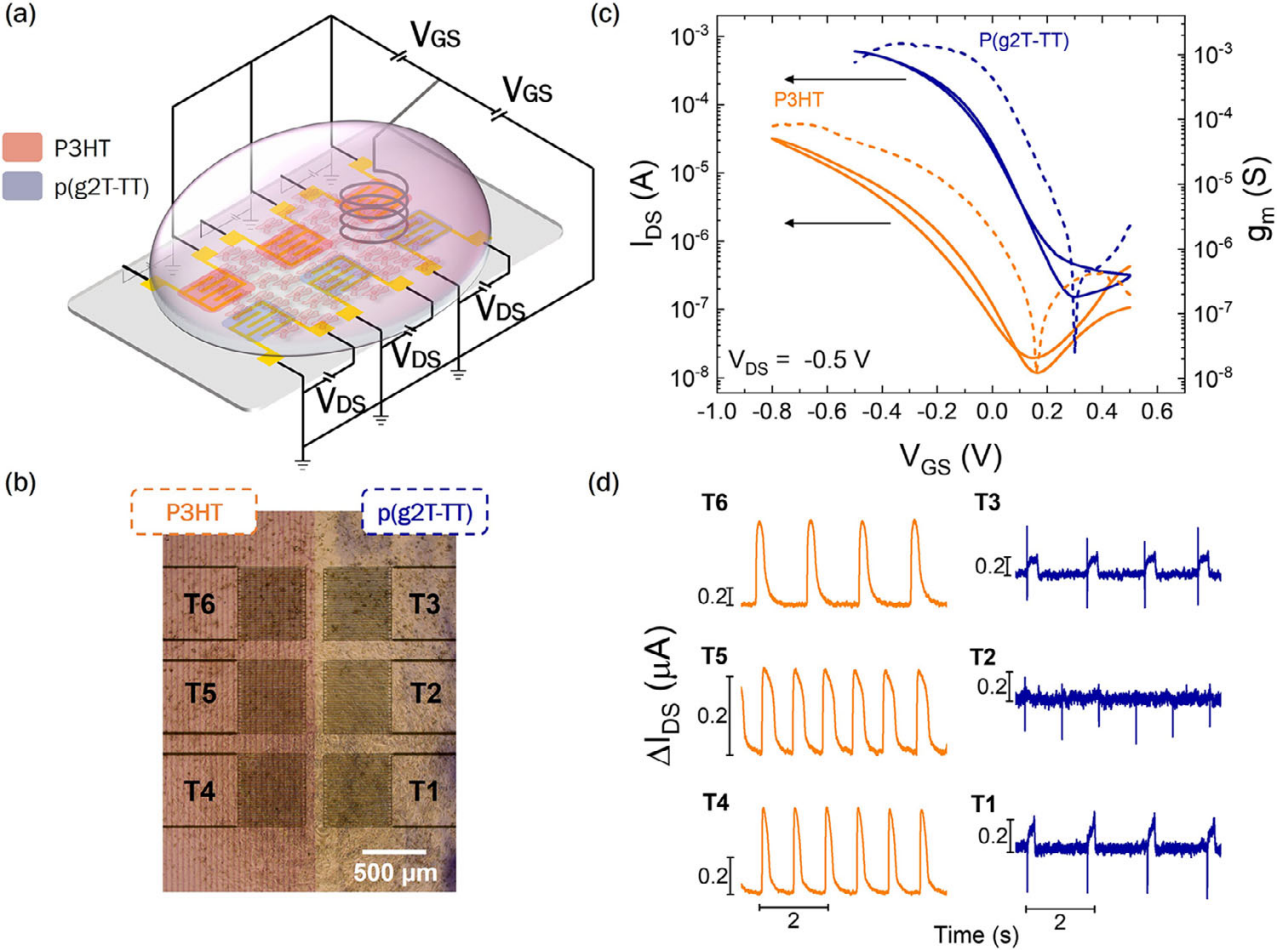
(a) Schematic representation of a 3 × 2 transistor array in which one half is printed with P3HT and the other with p(g2T‐TT). The entire array is then interfaced with the same cardiomyocyte monolayer. (b) Optical image of the EGOTs with plated cardiomyocytes on top of the two polymers printed side by side. The left three transistors, T4, T5, and T6, are printed with P3HT, right transistors, T1, T2, and T3, are printed with p(g2T‐TT). (c) Representative *I–V* curves of P3HT (orange) and p(g2T‐TT) (blue) EGOTs in the presence of hiPSC‐CMs. The gate voltage is swept from 0.5 to −0.8 V for P3HT devices and from 0.5 to −0.5 V for p(g2T‐TT) ones, while V_DS_ is set to −0.5 V. The transfer characteristics were acquired using a gate‐voltage sweep rate of approximately 0.83 mV/ms for P3HT‐based devices and approximately 1.25 mV/ms for p(g2T‐TT)‐based devices. g_m_ for both devices is reported as a dashed line. (d) AP and FP traces recorded from the sample depicted in (b). Sampling rate = 1 kHz. V_DS_ is set to −0.5 V, and V_GS_ is near the maximum transconductance for the device.

Given that p(g2T‐TT)‐based OECTs show a much larger *g*_m_ than P3HT‐based EGOFETs, the large variability and poor morphology of AP recordings with p(g2T‐TT) devices could be explained by a weaker coupling between p(g2T‐TT)‐based devices and the cells' electrical signals. To assess if this hypothesized weaker coupling is intrinsic to the different polymers adopted, and not a result of contingent conditions or biological variability, we designed a direct comparison experiment within the same 3×2 transistor array. In this configuration, p(g2T‐TT) was printed on top of 3 electrode pairs on one half of the array, while P3HT was printed on top of 3 different pairs on the other half, as schematically illustrated in Figure 3a. To reduce the biological variability, the same batch of hiPSC‐CMs was plated on the two types of EGOTs. This approach allowed the formation of a homogeneous monolayer of hiPSC‐CMs to interface simultaneously with both materials, leaving the polymer type as the only variable. The brightfield image in Figure 3b shows the 3 × 2 array configuration, with labelled interdigitated transistors (T1‐T6), 3 covered with P3HT (left, T4‐T6) and 3 with p(g2T‐TT) (right, T1‐T3), all bearing cardiomyocytes culture on top. Precise patterning was achieved using piezoelectric inkjet deposition, in conjunction with optimized inkjet parameters and substrate preprocessing protocols, resulting in a defined interface between the two materials. Both polymers were printed in a single layer. Representative transfer characteristic curves for P3HT and p(g2T‐TT)‐based EGOTs in the presence of the hiPSC‐CMs are reported in Figure 3c. At *V*_DS_ = −0.5 V, *I*_DS_ for P3HT devices is shown in orange, whereas that of p(g2T‐TT) devices is depicted in blue (left axis). The corresponding *g*_m_ for both devices is extracted and displayed as a dashed line (right axis). From a purely electrical perspective, we obtained a direct confirmation of the superior properties of p(g2T‐TT)‐based OECTs, where ions penetrate the bulk and modulate the entire channel conductivity [32], with respect to P3HT‐based EGOFETs, in which ionic modulation is largely confined to the interface [33]. p(g2T‐TT) devices exhibit higher *I*_DS_​ currents and markedly larger *g*_m_ values, reaching a few mS at their peak, over an order of magnitude larger than those of neighboring P3HT‐based EGOTs with identical geometry. Despite superior electronic performance on the same chip, the AP recordings shown in Figure 3d unambiguously confirm a striking contrast in signal quality, with much poorer recordings in the case of p(g2T‐TT). Devices with printed P3HT as active material consistently yield AP waveforms typical of ventricular cardiomyocytes as previously reported [14], whereas p(g2T‐TT)‐based OECTs produce attenuated and distorted signals, like previous observations in p(g2T‐TT) only arrays (Figure 2b).

Given the volumetric gating mechanism of OECTs, slower ionic‐electronic transport dynamics may, in principle, distort fast electrophysiological events such as the rapid upstroke phase (0) of cardiac APs. To assess whether the inferior AP fidelity observed with p(g2T‐TT)‐based OECTs originates from intrinsic device kinetics rather than cell‐polymer coupling, we quantitatively evaluated the temporal response of the devices in the absence of cells. We therefore measured the transient drain current response of p(g2T‐TT)‐based OECTs and P3HT‐based EGOFETs under identical operating conditions by applying a 5 ms square gate‐voltage pulse with an amplitude of 0.1 V, representative of the full AP swing of hiPSC‐CMs (Figure SI.6). P3HT‐based EGOFETs exhibited a rise time constant τ_rise_ = 0.28 ms, whereas p(g2T‐TT)‐based OECTs with identical geometry (*W* ∼ 2 cm, *L* = 10 µm) showed significantly slower kinetics (τ_rise_ = 1.1 ms), likely deriving from their volumetric capacitance. To determine whether this slower response was responsible for AP distortion, we optimized a p(g2T‐TT) OECT to obtain a faster transient (τ_rise_ = 0.3 ms), comparable to P3HT devices, by reducing its footprint (*W* = 100 µm, *L* = 10 µm) (see Figure SI.7) [34]. Faster p(g2T‐TT) OECTs, however, still failed to accurately reproduce AP morphology during cell recordings (Figure SI.7c).

### 2.3 Evaluation of hiPSC‐CM‐Substrate Coupling Through Immunofluorescence

These heterogeneous AP recordings between the two classes of polymeric EGOTs strongly point to a different cell‐polymer coupling, influenced by specific material physicochemical properties. P3HT films are hydrophobic, whereas p(g2T‐TT) films are hydrophilic [35], as confirmed by contact angle measurements (Figure SI.8). Moreover, topographic Atomic Force Microscopy (AFM) measurements reveal marked differences in the nanoscale surface roughness of the two polymer films. Printed p(g2T‐TT) films exhibit a local root‐mean‐square (RMS) roughness of 3.7–4.8 nm (representative device areas of 7.8 × 5.9 µm^2^ on the channel and 5.47 × 5.47 µm^2^ on the electrodes) (Figure SI.9), whereas printed P3HT films are significantly smoother, with local RMS roughness values of 0.7–0.9 nm (representative device areas of 18.7 × 9.4 µm^2^ and 2 × 2 µm^2^ on the channel) (Figure SI.10). Beyond these topographical differences, the presence of ethylene glycol side chains in p(g2T‐TT) may further modulate cell‐polymer coupling by reducing nonspecific protein adsorption at the cell‐device interface, a behaviour that has been widely reported for ethylene glycol‐based antifouling surfaces [36, 37, 38]. These differences in surface chemistry, morphology, and topography may influence cell‐substrate interactions, since they are known to affect adhesion protein synthesis, assembly, maturation, and disassembly, which eventually regulate cell shape, spreading, migration, differentiation, and, particularly for cardiomyocytes, can affect contractility and overall functionality [39]. Most importantly for electrical devices, cell adhesion determines the efficiency of the electrical coupling between cells and the underlying substrate [40, 41, 42]. A central feature of cell adhesion is the formation of focal adhesions (FAs), specialized structures that physically connect the cytoskeleton to the extracellular matrix (ECM). FAs are assembled from a complex network of proteins, including integrins, talin, paxillin, and vinculin. The latter plays a key role in cellular adhesion by regulating FA formation and turnover, and its expression is directly proportional to FAs size and density [43]. In addition, vinculin participates in controlling the mechanical properties of FAs, by regulating tension and stability, and their signalling capacity [44]. As a cytoskeletal protein, vinculin interacts with both actin filaments and integrin receptors, thereby linking the ECM to the intracellular cytoskeleton [45], and it is involved in the transmission of mechanical forces between the cell and the ECM, enabling cells to sense and adapt to substrate stiffness and topography [46]. In the case of cardiomyocytes, the role of vinculin in cytoskeletal dynamics is pivotal for maintaining the functionality of cardiac tissue. A loss of vinculin in myocardium was reported to provoke the destabilization of gap and adherent junctions, costameres, resulting in an inadequate electrical and mechanical coupling [47]. Conversely, enhanced vinculin expression was associated with stronger adhesion on exogenous scaffolds in vitro, which in turn led to optimized hiPSC‐CMs properties, including better organization, maturation, contraction [48, 49], and promoted myofibril maturation [50].

**FIGURE 4:**
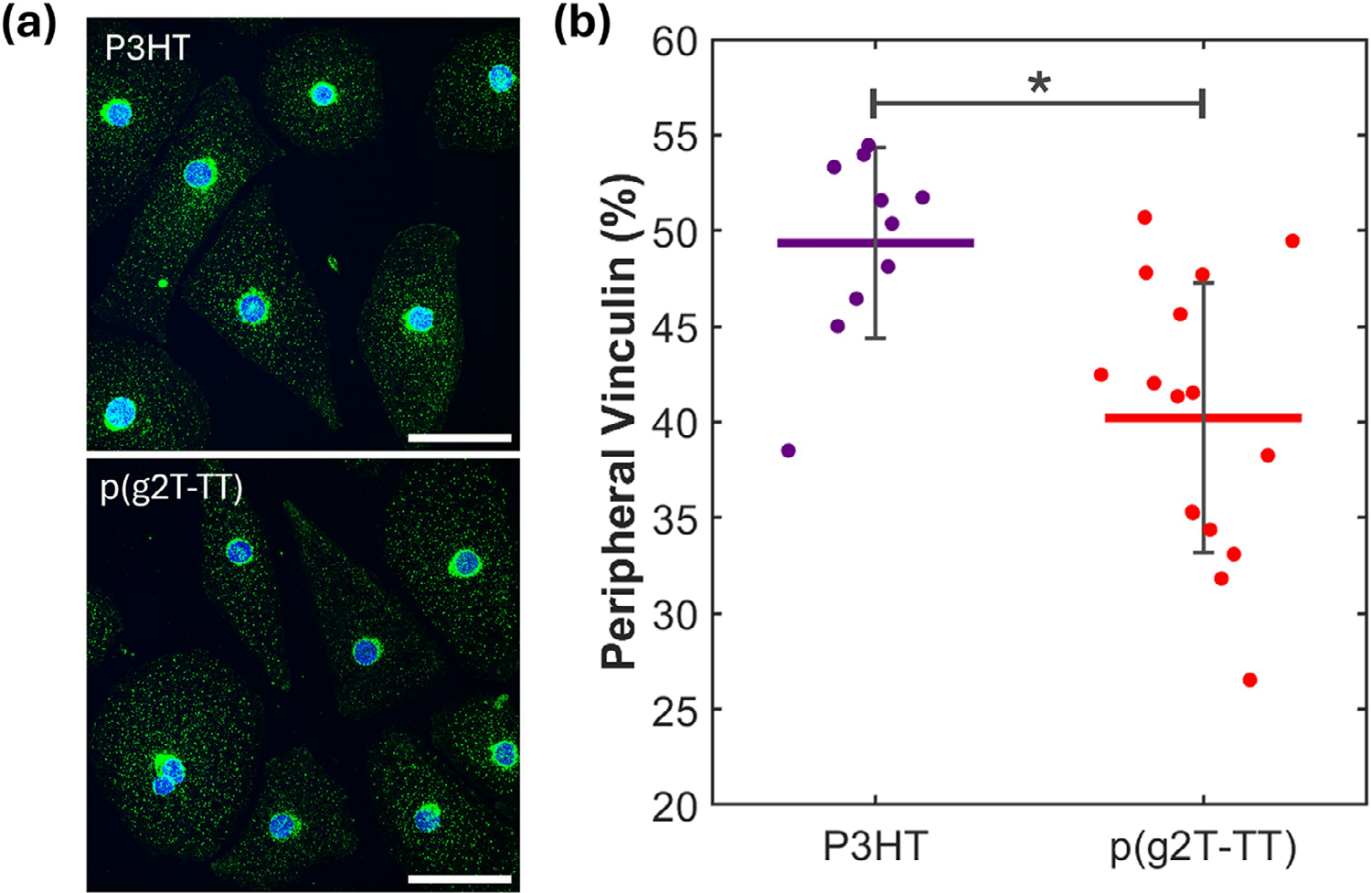
(a) Representative immunofluorescence images of hiPSC‐CMs labelled with vinculin antibody (green) and Hoechst (nuclei, blue). Scale bars: 50 µm. (b) Quantitative analysis of the percentage of vinculin area in the cell peripheral region, in the case of hiPSC‐CMs cultured on top of P3HT and p(g2T‐TT) thin films. Statistical analysis performed with Student's *t*‐test, p‐value: ^*^ *p* <0.05. N = 10 for P3HT samples, N = 16 for p(g2T‐TT) samples.

To investigate the differences in hiPSC‐CM adhesion on P3HT and p(g2T‐TT) substrates, we analyzed vinculin expression by immunofluorescence (Figure 4). This method is widely employed to evaluate cell‐substrate adhesion in both 2D and 3D scaffolds and has been applied to various cell types, including osteocytes [51], fibroblasts [52], endothelial cells [53], embryonal carcinoma cells [54], and hiPSC‐CMs  [55, 56]. For each condition (P3HT and p(g2T‐TT) printed on glass coverslips), we quantified the vinculin‐covered area in N = 40 cells, obtained respectively from 10 confocal scans for P3HT and 16 scans for p(g2T‐TT). These datasets were collected from two independent biological differentiation runs, each comprising three experimental replicates per substrate, to adequately capture inter‐sample variability. Optical sections spanning the entire cell volume were acquired, and the vinculin‐positive area was quantified from z‐projection images. The representative images reported in Figure 4a show vinculin clusters distributed in the whole cell area, with larger aggregates present in the perinuclear region. These aggregates correspond to inactive cytoplasmic vinculin, not directly related to cell‐substrate adhesion [57, 58, 59, 60]. Under poor adhesion conditions, vinculin is known to be localized almost exclusively in these perinuclear pools [51, 61]. To distinguish perinuclear from peripheral vinculin, we created a separate mask for each cell based on the Hoechst channel (see Experimental Section for further details) and expanded each mask by 2 µm, thus defining a per‐cell perinuclear region to be excluded from quantification. This distance was selected as a minimal buffer region that reliably excludes the perinuclear cytoplasmic compartment, which is known to contain predominantly inactive vinculin pools not engaged in adhesion structures. Moreover, FAs are consistently reported to form preferentially at the distal cell edge and to be largely absent within the first few micrometers surrounding the nucleus, due to the local cytoskeletal architecture and low traction forces near the nuclear envelope [59, 60, 62, 63]. For these reasons, the enlarged nuclear area was excluded from quantification. The remaining portion of the cell area, i.e., the cytoplasmic region outside the expanded nuclear mask, was defined as the peripheral region, corresponding to the zone where adhesion‐associated vinculin accumulates and where FAs typically form along the cell boundary. A detailed schematic of this segmentation workflow is provided in the image‐processing flow chart in the Supporting Information (Figure SI.11) and further described in the experimental section. Through the analysis of the sum‐intensity projections of the acquired images (Figure 4a), we found a significantly higher percentage of peripheral vinculin in the P3HT case (Figure 4b), thus highlighting an improved adhesion of cells in the presence of P3HT with respect to the p(g2T‐TT) case. While this analysis does not directly probe differences in the electrical properties of the cell‐device cleft, these findings are consistent with the superior performance of P3HT‐based EGOFETs in recording high‐fidelity action potentials, likely reflecting more effective cell‐polymer coupling.

## 3 Conclusions

Our work demonstrates that the interplay between intrinsic electrical performance and cell‐device coupling is crucial for reliably transducing bioelectronic signals. While polymers like p(g2T‐TT) are attractive candidates for biosensing due to their superior electrical performance and good biocompatibility, these properties alone do not guarantee effective coupling with the electrical activity of cells. Indeed, we show that the intrinsic electrical performance of polymeric EGOTs does not directly translate into signal fidelity. By employing p(g2T‐TT)‐based OECTs to record APs from hiPSC‐CMs, we found that although they outperform P3HT‐based EGOFETs in terms of transconductance, only a small fraction of the recorded signals exhibited the characteristic AP morphology. The majority instead displayed FP‐like waveforms with marked heterogeneity. Such evidence indicates that recording fidelity, which requires sufficient transconductance, is also governed by biological interactions at the cell‐polymer interface, playing a key role in the electrical coupling. The direct, one‐to‐one comparison between P3HT and p(g2T‐TT) device behavior that we have reported underscores the role of material physicochemical properties in shaping cell‐device surface interactions. Increased peripheral localization of vinculin in cells on P3HT, typically associated with stronger focal adhesions, is expected to improve cell‐substrate interaction and enhance cleft resistance, ultimately favoring higher‐quality signal transduction. Together, these findings highlight the need to move beyond purely electronic figures of merit when evaluating materials for bioelectronics. Future optimization of bioelectronic interfaces will require an integrated approach that combines materials engineering with an understanding of cell adhesion biology, in order to achieve both high electrical performance and reliable biological coupling.

## 4 Experimental Section

### 4.1 Electrodes Fabrication

EGOT devices were fabricated on 700 µm‐thick Corning glass substrates, chosen for their mechanical durability and compatibility with cardiomyocyte cultures. The patterning of source and drain electrodes was accomplished via a maskless image reversal lithography method. Following oxygen plasma treatment, a layer of AZ5214E photoresist was applied by spin‐coating at 4000 rpm for 60 s and subsequently soft‐baked at 110 °C for 90 s to facilitate solvent evaporation. The resist‐coated substrates were then negatively exposed using a Heidelberg MLA100 direct‐write lithography system. A second bake at 120 °C for 90 s preceded flood UV exposure, completing the image reversal and enabling selective development of the pattern in AZ726 MIF.

Metal contacts were deposited by thermal evaporation of a 2 nm chromium adhesion layer, followed by 30 nm of gold. The final electrode geometry was defined by lift‐off in Technistrip Micro‐D2, yielding interdigitated source‐drain structures with channel dimensions of 10 µm in length and ∼20,000 µm in width. Custom‐made wells were used to confine the cell culture area on top of the devices. The wells were fabricated by 3D printed PLA and mounted on the devices using a biocompatible adhesive (RS 187‐3460) (see Figure SI.12 for a sketch of the device).

### 4.2 p(g2T‐TT) Inkjet Printing

To prevent the gold leads from being exposed to the electrolyte, some parts of the transistors, except the channel area, were coated with a biocompatible insulating material (SU8‐TF6001) using a Fujifilm Dimatix DMP‐2831 inkjet printer. The SU8 insulation layer thickness, measured by AFM line profiling across a scratched substrate reference, is approximately 5.45 µm (Figure SI.13). The insulating material pattern was first baked at 120 °C to evaporate the solvent, followed by UV exposure for 15 min and a subsequent annealing at 120 °C for 3 min. Fujifilm Dimatix DMP‐2831 was also used for the printing of the polymers. The p(g2T‐TT)‐semiconducting ink was prepared by dissolving the polymer in ortho‐dichlorobenzene at a concentration of 1.5 mg/mL. The solution was stirred at 90 °C for several hours to ensure complete dissolution and homogeneity. Inkjet printing was carried out at room temperature using a Dimatix printer, with a drop spacing of 15 µm. Post‐printing annealing was carried out at 120 °C for 1 h in an ambient environment. The obtained film thickness is approximately 36 nm (see Figure SI.9 panel f‐g). These optimized conditions were found to yield high‐quality films with desirable electronic properties for device performance.

### 4.3 P3HT Inkjet Printing

After the application of the abovementioned insulating material, the semiconducting layer of P3HT, a commercially available high‐purity regioregular polymer, was deposited via inkjet printing onto the transistor channel regions. A custom‐formulated ink composed of P3HT dissolved in a binary solvent system of chlorobenzene and ortho‐dichlorobenzene in a ratio of 3:1 with a concentration of 2.6 mg/mL was prepared. Printing was carried out with the substrate heated to 35 °C and a drop spacing of 45 µm. The annealing of the films was carried out at 120 °C overnight in a nitrogen environment. This specific formulation and set of parameters were selected to ensure consistent film formation, high current modulation (on/off ratio), minimal hysteresis, and reliable sample reproducibility.

### 4.4 hiPSC Culture and Differentiation Toward hiPSC‐CMs

Wild‐type human induced pluripotent stem cells (hiPSCs) were obtained from the Coriell Institute for Medical Research (identifier GM25256; WTC‐11 line, hPSCReg UCSFi001‐A) under the appropriate Material Transfer Agreement. Cells were maintained on multiwell plates coated with recombinant human vitronectin (rhVTN, Thermo Fisher Scientific) in E8 Flex medium (Thermo Fisher Scientific). Prior to cardiac differentiation, hiPSCs were replated on hESC‐qualified Matrigel (BD Biosciences). Differentiation into cardiomyocytes (hiPSC‐CMs) was carried out using a GSK3βB/Wnt‐signaling modulation protocol as previously described [64] with minor modificationsonto Matrigel‐coated plates (Corning) [64]. On day 0, cells were treated with 6 µM CHIR99021 in RPMI 1640 medium (Euroclone) supplemented with B27 minus insulin (Thermo Fisher Scientific). On day 1, the culture volume was topped up with 67% fresh RPMI/B27 minus insulin. On day 2, an additional 33% fresh medium (relative to the initial day 0 volume) was added. On day 3, the medium was changed to RPMI/B27 minus insulin containing 5 µm IWR‐1 for 24 h. On day 5, the medium was replaced with RBK1 medium consisting of RPMI 1640 medium (Euroclone) supplemented with 1X B27 Supplement (Thermo Fisher Scientific) and 1% KnockOut Serum Replacement (Thermo Fisher Scientific) for two days [65]. Following differentiation, hiPSC‐CMs were purified by glucose starvation, yielding a final purity >90% hiPSC‐CMs [66]. Cells were cryopreserved in Bambanker (Nippon Genetics) between day 9 and day 16 of differentiation. For subsequent experiments, cryopreserved hiPSC‐CMs were thawed, replated at low density and expanded in RPMI/B27 supplemented with 1% KOSR and 4 µM CHIR99021 (SelleckChem), as previously described [65]. For the hiPSC‐CM maintenance, RBK1 medium was used.

### 4.5 Viability, Proliferation, and Live/Dead Assay

Human induced pluripotent stem cell‐derived cardiomyocytes (hiPSC‐CMs) were seeded on human fibronectin‐coated P3HT and p(g2T‐TT) substrates, previously prepared via Inkjet printing (sample preparation details described above), at a density of ∼5 × 10^5^ cells/cm^2^. Cell proliferation was evaluated at 96 and 168 h post‐plating on the polymers deposited on glass coverslips in 12‐well plates. Prior to each measurement, the culture medium was refreshed with a new medium containing 100 mg/mL AlamarBlue (Thermo Fisher). AlamarBlue contains resazurin, which is reduced to the fluorescent resorufin within viable cells, serving as an indicator of cell viability and proliferation. Samples were incubated for 3 h at 37°C under 5% CO_2_ in the dark. Afterward, three 100 µL aliquots of culture media per sample were transferred to black 96‐well microplates, and fluorescence was measured using a TECAN Spark microplate reader (excitation: 530 nm, emission: 590 nm). A live/dead viability assay was performed on day 7 using the ReadyProbes Cell Viability Imaging Kit (Blue/Red; Thermo Fisher). This kit employs NucBlue Live (Hoechst 33342) to stain all nuclei (detected using a DAPI filter configuration; excitation/emission maxima: ∼394/460 nm) and NucRed Dead to label only nuclei of cells with compromised membranes (detected using an RFP filter set; excitation/emission maxima: ∼545/630 nm). Three samples were stained per condition, and for each sample, five images were acquired. Imaging was carried out with an upright Olympus BX63 microscope, equipped with a 20× water‐immersion objective, a Crest Optics X‐Light V2 spinning disk confocal module, and a Teledyne Photometrics Prime BSI sCMOS camera. To count cell nuclei, images were processed in Fiji (ImageJ). A threshold was first applied to generate a binary mask, followed by particle analysis to identify and quantify nuclei.

### 4.6 Сell Plating on the Devices

Semiconductor devices were sterilized by sequential rinses with sterile water, 70% ethanol, and 100% ethanol (two times), followed by air‐drying for ∼5 min under a laminar flow hood. The channel area was subsequently coated with a 40 µg/mL bovine fibronectin (Merck) solution prepared from a 1 mg/mL stock in PBS, Ca^2+^, and Mg^2+^ free. A volume of 8 µL per well was used for standard chips, while larger surfaces were coated with proportionally higher volumes. All coated devices were placed in a humidified Petri dish to minimize evaporation and incubated at 37 °C for 40–60 min. After incubation, the fibronectin solution was carefully aspirated, and cells were seeded immediately to prevent drying of the coated surface. hiPSC‐CMs were seeded at a density of ∼ 5 × 10^5^ cells/cm^2^. For each device, cells were applied in 10 µL drops of RBK1 medium supplemented with Revitacell (Thermo Fisher Scientific) directly onto the coated transistor surface. The chips were incubated for 1 h at 37 °C, 5% CO_2_ to allow formation of a uniform cell monolayer. Following attachment, 300 µL of RBK1 medium supplemented with Revitacell was gently added to each chamber. The next day, and subsequently twice per week, ∼60% of the medium volume was refreshed with a pre‐warmed RBK1 medium.

### 4.7 AP Recordings via EGOTs

Electrical measurements and action potential (AP) recordings of EGOTs were performed using an Agilent B2912A precision source‐measure unit. Before initiating biological recordings, devices plated with hiPSC‐CMs were first assessed by acquiring transfer characteristics. In this step, the gate voltage (*V_GS_*) was linearly swept from +0.5 to −0.8 V in P3HT‐based devices and from +0.5 to −0.5 V in p(g2T‐TT)‐based ones, while maintaining the drain‐source voltage (*V_DS_*) constant at −0.5 V. The transconductance (*g_m_*) was determined by numerically deriving the *I_DS_‐V_GS_* curve, calculated as *g_m_* = ∂I_DS_/∂V_GS_. A platinum wire (99.99+% purity, 0.5 mm diameter, Advent Research Materials) served as the gate electrode. To capture action potentials, time‐resolved drain current (*I*_*DS*_(*t*)) signals were recorded under fixed *V_GS_* and *V_DS_* conditions, with a temporal resolution of 1 kHz. Post‐processing of the acquired current traces involved baseline correction to eliminate low‐frequency drift. This was achieved using Origin 2018's Peak Analyzer tool, applying the “Asymmetric Least Squares Smoothing” method under the Baseline Mode function. All measurements were performed outside the incubator. To maintain physiological conditions, the cells were kept at 37 °C using a precision heating stage (Linkam LTS420).

### 4.8 Contact Angle Measurements

Surface wettability and hydrophilicity of the polymer films were evaluated by static water contact angle measurements using the sessile drop method (OCA‐15, Data Physics). A 5 µL Milli‐Q water drop was dispensed on the polymer films, previously inkjet printed on Corning glass substrates. The angle was evaluated from N = 9 P3HT‐coated glasses and N = 10 p(g2T‐TT)‐coated glasses.

### 4.9 Atomic Force Microscopy (AFM)

Atomic force microscopy (AFM) imaging was performed using a Bruker Dimension Icon XR AFM system operated in ScanAsyst mode under ambient conditions. Measurements were carried out using SCANASYST‐AIR‐HPI silicon nitride cantilevers. Height (topography) images were acquired at multiple locations across the device surface, including the polymer channel, electrode regions, and scratched substrate references used for thickness determination. Scratches were manually created using a tungsten tip to expose the underlying substrate for step‐height and thickness measurements prior to AFM imaging. AFM data processing and image flattening were performed using Gwyddion software. Line‐by‐line and plane flattening were applied with an appropriate mask to remove background tilt and scanner bow without altering local surface features. Root‐mean‐square (RMS) roughness values were extracted from representative regions of interest after flattening.

### 4.10 Cell Morphology Study and Immunofluorescence Staining

After 7 days of culture, cells were stained with NucBlue Live reagent (Hoechst 33342) and ActinGreen 488 ReadyProbes Reagent (Molecular Probes) to evaluate nuclear morphology and actin filaments. Prior to actin staining, cells were washed three times with PBS, fixed with Antigenfix solution (Diapath), rinsed, permeabilized with 0.1% Triton X‐100 (Sigma–Aldrich), and washed three times with PBS. Vinculin expression was analyzed at day 4 post‐plating. Cells were washed with PBS, fixed in Antigenfix solution (Diapath), permeabilized for 10 min in 0.1% Triton X‐100 in 1% PBS, and washed twice with PBS. Blocking was performed using 5% bovine serum albumin (BSA; Sigma–Aldrich) in 1% PBS for 1 h at room temperature. Samples were then incubated overnight at 4°C with rabbit anti‐vinculin primary antibody (Thermo Fisher Scientific, lot no. 3005441) at a 1:500 dilution in 1% BSA/PBS. Following two washes in PBS containing 0.05% Tween‐80 (PBST), samples were incubated for 2 h at room temperature with Alexa Fluor 488 goat anti‐rabbit IgG (H+L) cross‐adsorbed secondary antibody (Thermo Fisher Scientific, lot no. 2873188) at 1:500 in 1% BSA/PBS. After three PBST washes (5 min each), nuclei were counterstained with Hoechst 33342 (1 µg/mL in PBS) for 10 min at room temperature in the dark, followed by two PBS washes. Samples were stored in PBS at 4 °C until imaging.

### 4.11 Confocal Microscopy and Focal Adhesion Analysis

Confocal imaging for actin and vinculin was performed on a Nikon Eclipse Ti2 inverted confocal laser scanning microscope (Nikon Instruments), equipped with a 60× oil immersion objective, laser lines at 403, 487, 561, and 636 nm, and emission filter sets at 450/50, 525/50, 595/50, and 700/75 nm. Excitation and emission conditions for each fluorophore are summarized in Table 1:

**TABLE 1:** Excitation Laser and emission filters employed in fluorescence imaging experiments.

Fluorescent probe	Laser source (nm)	Emission filter (nm)
Hoechst 33342	403	450/50
ActinGreen 488	487	525/50
Alexa Fluor 488	487	525/50

For vinculin coverage analysis, between 10 and 16 scans were conducted for each condition across three experimental replicates per substrate and two differentiations. Image analysis was performed with Fiji (ImageJ). Z‐stack images from both the Hoechst and vinculin channels were converted into sum‐intensity projections. Nuclei were segmented from the Hoechst nuclear channel to generate nuclear regions of interest (ROIs), which were then dilated concentrically by 2 µm to create expanded perinuclear ROIs for each nucleus. In the vinculin channel, a threshold (default method) was applied to the z‐projection to quantify the total vinculin‐positive area. The expanded nuclear ROIs were overlaid onto the thresholded vinculin projection to measure the amount of vinculin signal within the perinuclear region. The perinuclear vinculin percentage was calculated as the ratio between perinuclear vinculin and total vinculin signal, while the peripheral vinculin percentage was obtained by subtracting the perinuclear percentage from the total (see Figure SI.11 for details on the image processing pipeline).

## Author Contributions

G.Z. designed the electrical recording experiments supervised by A.K. and M.C. E.M. designed the immunostaining experiments supervised by M.R.A. G.Z. fabricated the EGOTs. C.B. formulated the p(g2T‐TT) ink. G.Z. and C.B. printed p(g2T‐TT) devices. hiPSC‐CMs were cultured and plated in P.J.S.’s lab by L.S., and A.Kh. L.S. coordinated all the parts related to cell culture. G.T. and E.M. carried out viability assays and cell immunostaining experiments under the supervision of M.R.A. S.T. performed AFM measurements, data analysis, and interpretation. G.Z. carried out all EGOT recordings. G.Z. and E.M. carried out the data analysis. G.Z. wrote the manuscript with input from all the authors. M.R.A., A.K., and M.C. supervised and managed the project.

## Conflicts of Interest

The authors declare no conflict of interest.

## Supporting information




**Supporting File**: advs74136‐sup‐0001‐SuppMat.docx.

## Data Availability

The data that support the findings of this study are available from the corresponding author upon reasonable request.
