# Supplementary material for: Beyond Transconductance: Cell‐Polymer Coupling Determines Fidelity in Action Potential Recording via Electrolyte‐Gated Polymer Transistors
Source: Adv Sci (Weinh). 2026 Feb 12;13(30):e20122. doi: 10.1002/advs.202520122 (PMC13248828; doi:10.1002/advs.202520122)
Supplement: Supplementary file 1 — Supporting File: advs74136‐sup‐0001‐SuppMat.docx. [file ADVS-13-e20122-s001.docx]

***Supporting Information***

**Beyond Transconductance: Cell-Polymer Coupling determines Fidelity in Action Potential Recording via Polymer Transistors**

Giulia Zoe Zemignani^1¥^, Elena Mancinelli^1¥^, Gabriele Tullii^1^, Aleksandr Khudiakov^2^, Cristiano Bortolotti^1,3^, Shubham Tanwar^1^ Peter J Schwartz^2^, Luca Sala^2,4^, Maria Rosa Antognazza^1*^, Mario Caironi^1*^, Adrica Kyndiah^1*^

1. Center for Nano Science and Technology, Istituto Italiano di Tecnologia, Via Rubattino 81, 20134 Milano, Italy
2. Istituto Auxologico Italiano IRCCS, Center for Cardiac Arrhythmias of Genetic Origin and Laboratory of Cardiovascular Genetics, Milan, Italy
3. Department of Electronics, Information and Bioengineering, Politecnico di Milano, Piazza Leonardo da Vinci, 32, 20133 - Milano, Italy
4. Department of Biotechnology and Biosciences, University of Milano-Bicocca, Milan, Italy

Correspondence to: [mariarosa.antognazza@iit.it](mailto:mariarosa.antognazza@iit.it); [mario.caironi@iit.it](mailto:mario.caironi@iit.it); [adrica.kyndiah@iit.it](mailto:adrica.kyndiah@iit.it)

^¥^ Equal Contributions

**SI.1) Design of the Electrodes**

The “standard design” corresponds to the P3HT-based EGOFET architecture employed in our previously published work ^[1]^. The “small design” refers to the optimization of the temporal characteristics of p(g2T-TT) OECTs, as detailed in the following, Figure SI.6.


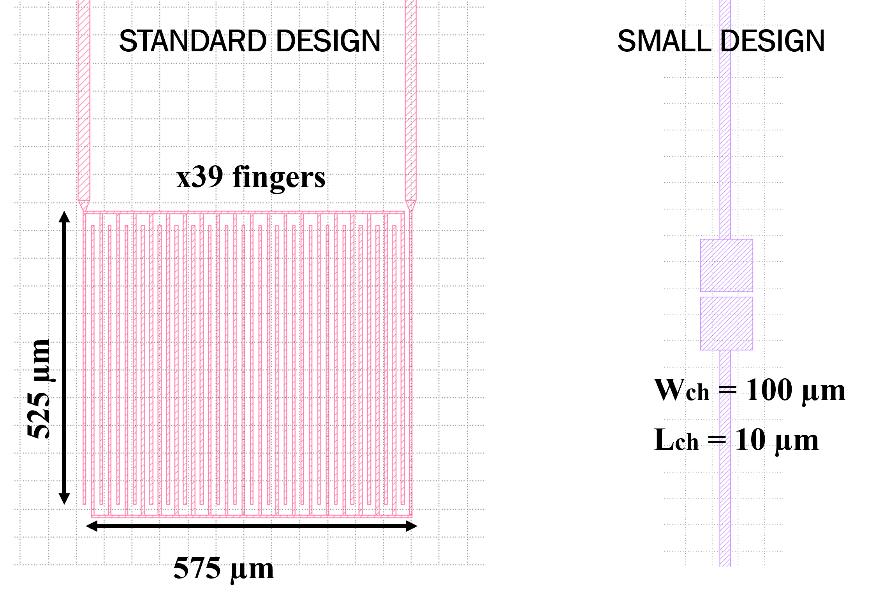


**Figure SI.1**: Design of the electrodes. Left: standard interdigitated design consisting of 39 fingers (W ~ 2 cm, L = 10 µm). Right: small single-channel design with W_ch_ = 100 µm and L_ch_= 10 µm.

**SI.2) Live/Dead Assay**

In addition to the Alamar Blue assay, a live/dead assay was conducted to further assess the long-term viability of cells cultured on p(g2T-TT) films. After 7 days of incubation, cells were stained with Hoechst and NUC Red Dead 667 ready probes and subsequently analyzed. As shown in **Figure** **SI.2**, 98% of the cells remained viable on the p(g2T-TT) substrates (N = 15 images across three experimental replicas per substrate).

**
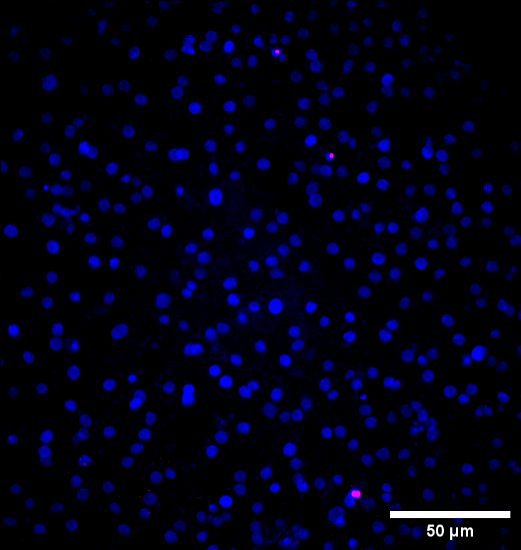
**

**Figure SI.2:** Viability of hiPSC-CMs evaluated with the live/dead assay 7 days after plating. The nuclei of all cells and of dead cells were stained with Hoechst (blue) and NUC red dead 667 ready probes (red), respectively.

**SI.3) Instability in AP Signal Transduction in p(g2T-TT)-based OECTs**

In the few instances where effective cell-polymer coupling was achieved, an initial AP waveform was recorded using p(g2T-TT)-based OECTs; however, this signal gradually diminished over time, eventually resembling an FP-like signal. This behavior indicates that the interface lacks stability, particularly when compared to P3HT-based devices, which consistently recorded stable AP waveforms for up to 30 minutes ^[1]^.


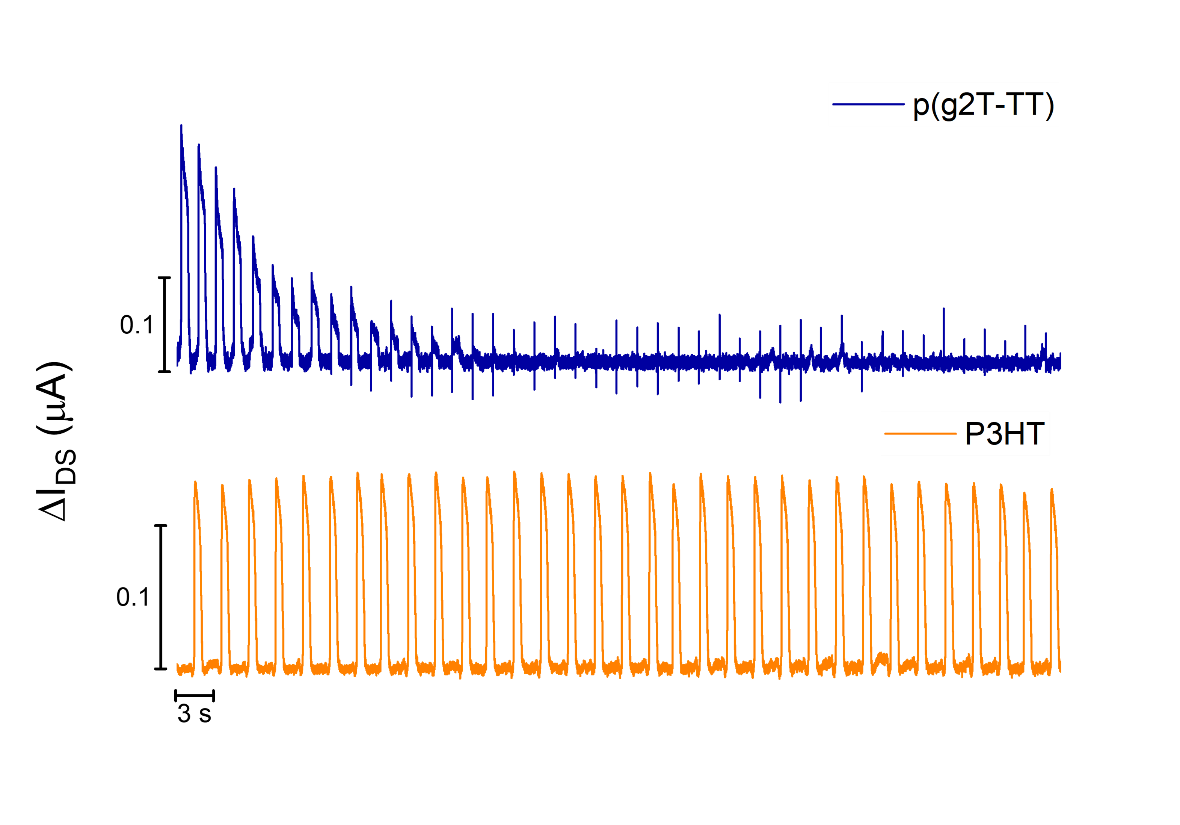


**Figure SI.3**: hiPSC-CMs signal recording using p(g2T-TT)-based OECTs (top) and P3HT-based EGOFETs (bottom).

**SI.4) Different FP-like Morphologies Recorded in the Experiments**

Depending on the coupling between the cell and the p(g2T-TT) channel, the morphology of FP-like signals can vary not only from sample to sample but also within the same device. For instance, in the figure below, the dashed boxes highlight AP recordings from transistors within the same array and cell culture, yet exhibiting distinct waveform shapes.


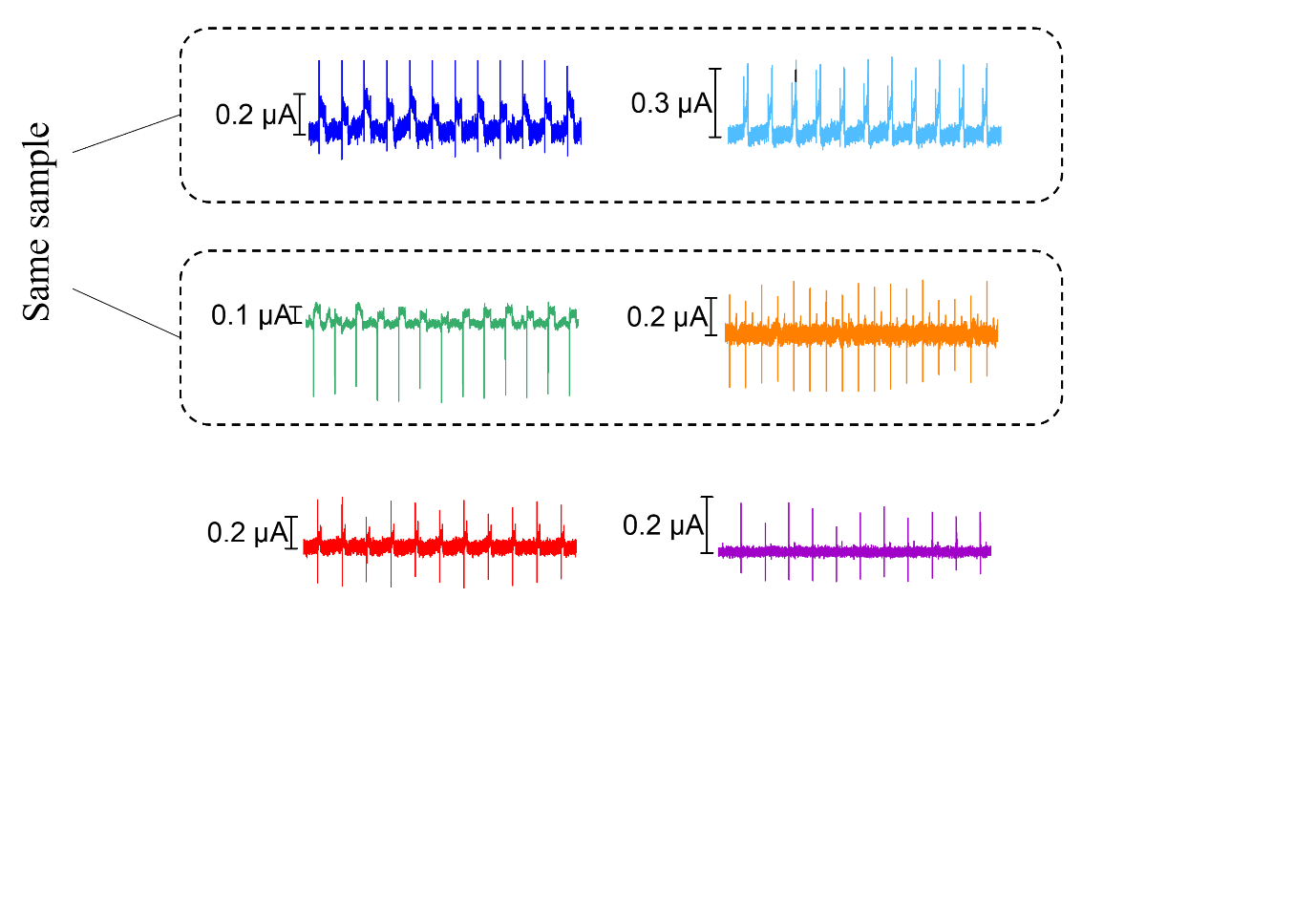


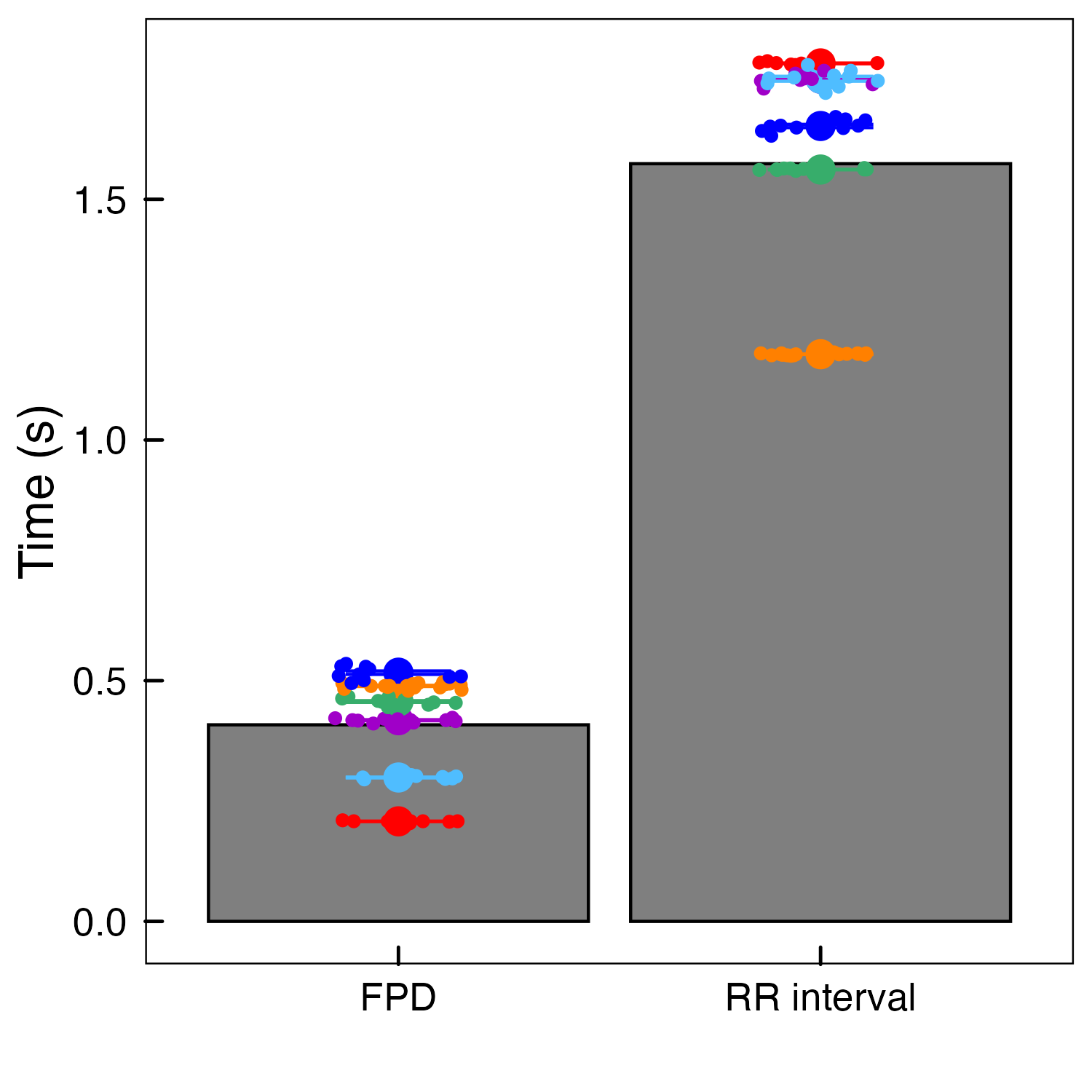


**Figure SI.4**: Examples of recorded FP-like traces having different morphologies. The first and the second row contain recordings belonging to the same culture well, hence the same array of transistors. The bottom panel shows quantification of key temporal parameters such as the FP duration (FPD), defined as the time interval between the depolarization peak and the repolarization phase of the FP, and the RR interval, defined as the time between two consecutive FP peaks. Each small dot represents the value for each FP, with colors matching the respective trace in the above panel. Large dots indicate mean values for each trace. Bars represent the mean of all datapoints. Error bars are not visible as smaller than the size of the mean dot.

**SI.5) Quantification of Electrophysiological Parameters from Samples with Efficient or Inefficient Coupling**

We performed a quantitative analysis of signal morphology for both AP-like (efficient coupling) and FP-like (inefficient coupling) recordings shown in main **Figure 2**, comparing the main electrophysiological temporal parameters that could be extracted from single events in each condition. AP-like events are extracted individually from the traces from main **Figure 2A** (**Figure SI.5(a-b)**). AP durations (APD) are calculated at different percentages of repolarization (20%, 50%, 90%) from the AP voltage peak. FP-like signals are extracted individually from both examples in main **Figure 2B** (**Figure SI.5(c-e)**)

Raw traces were exported as comma separated value files (.csv), converted to Axon Text File (.atf) format. The analyses of the AP traces and FP-like signals were performed in ClampFit (v. 11.2.2) following a previously published protocol^[2]^. Data were averaged and plotted using R (v.4.3.2) and ggplot2 ^[3]^.


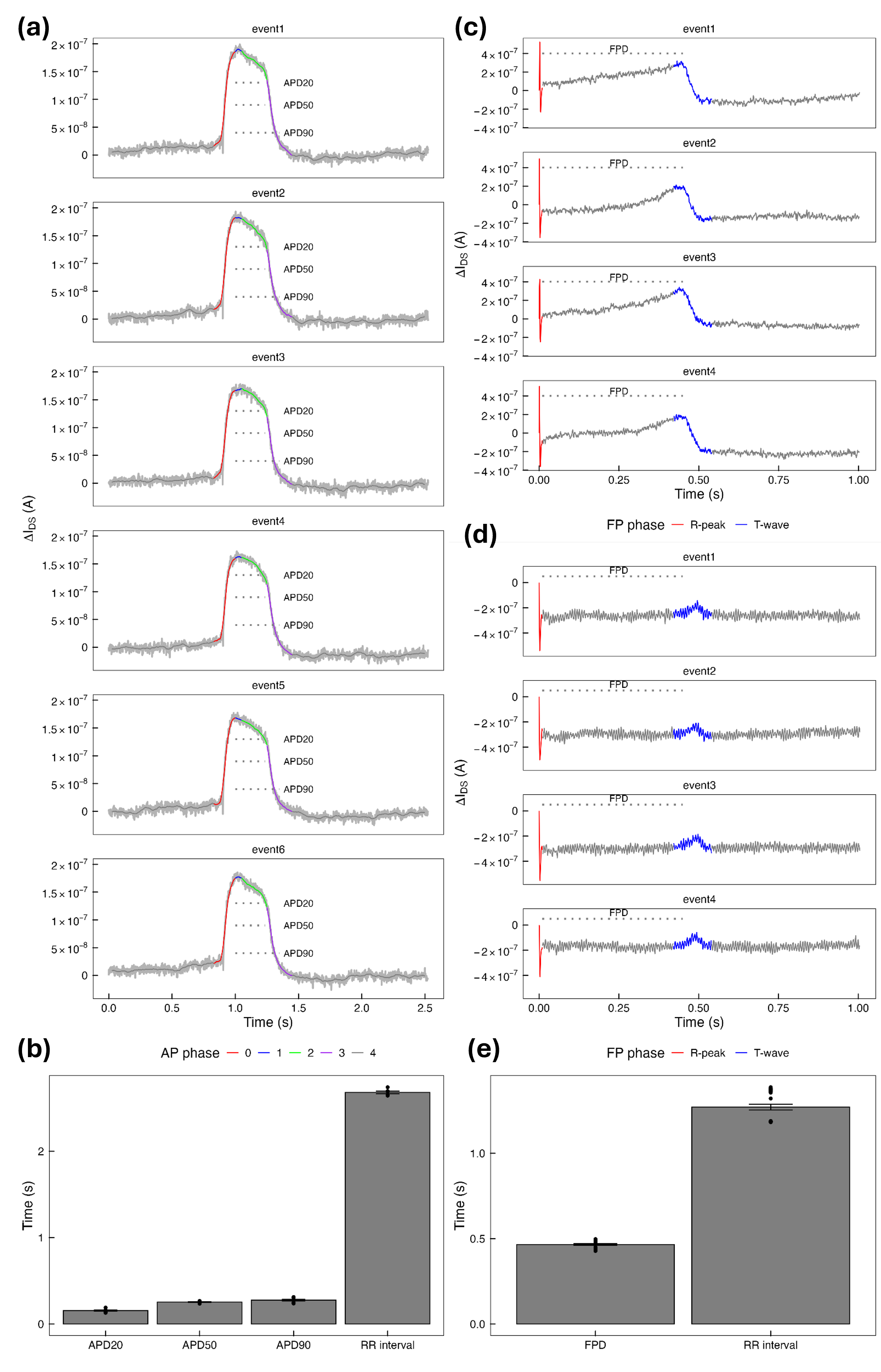


**Figure SI.5**: (a) Individual analysis of each AP-like event, with common AP phases explicitly color-coded. This specifically allows in-depth investigations of AP morphologies potentially related to drug effects or disease phenotypes. (b) Mean temporal data extracted from the events in A. (c-d) Individual analysis of four selected FP-like events, with traditional depolarization (red) and repolarization (blue) regions color-coded. (e) Mean temporal data extracted from events in (c-d). As visible, a more accurate in-depth quantification of temporal parameters during the whole electrical cardiomyocyte cycle is possible only when efficient coupling (i.e. AP-like signals) is achieved.

**SI.6) Time Response of P3HT and p(g2T-TT)-based EGOTs in Cardiac Cell Medium**

Time response measurements for the two selected polymers, P3HT and p(g2T-TT), were carried out using devices with identical standard geometries (*W* ~ 2 cm, *L* =10 μm), as shown in **Figure SI.6(a, b)**. Additional measurements were performed on p(g2T-TT)-based devices with reduced channel dimensions (*W* = 100 μm, *L* = 10 μm), as shown in **Figure SI.6(c)**. All devices were operated in the same cell medium electrolyte (RBK1). The time response curves were acquired after the application of a 5 ms squared pulse of -0.1 V (aperture time 0.1 ms) to the gate electrode of the EGOTs representing the transient behaviour of the devices without cells plated on top. For each *W/L* architecture, the applied *Vgs* was selected within the range of maximum transconductance of the corresponding device. This choice ensured consistency with the *V*gs values used during hiPSC-CM recordings. The 0.1 V pulse amplitude was chosen to match the full action potential amplitude of the hiPSC-CMs, ensuring physiological relevance while probing the dynamic response of the devices.

As expected, P3HT-based EGOFETs with standard geometry show a fast time response with τ_rise_ = 0.28 ms, whereas the equivalent p(g2T-TT) geometry device shows slower transients of τ_rise_ = 1.1 ms. The reduced dimension of the p(g2T-TT)-based OECT in **Figure SI.6(c)** shows, as expected, an improved τ_rise_ = 0.3 ms, in the range of P3HT devices.


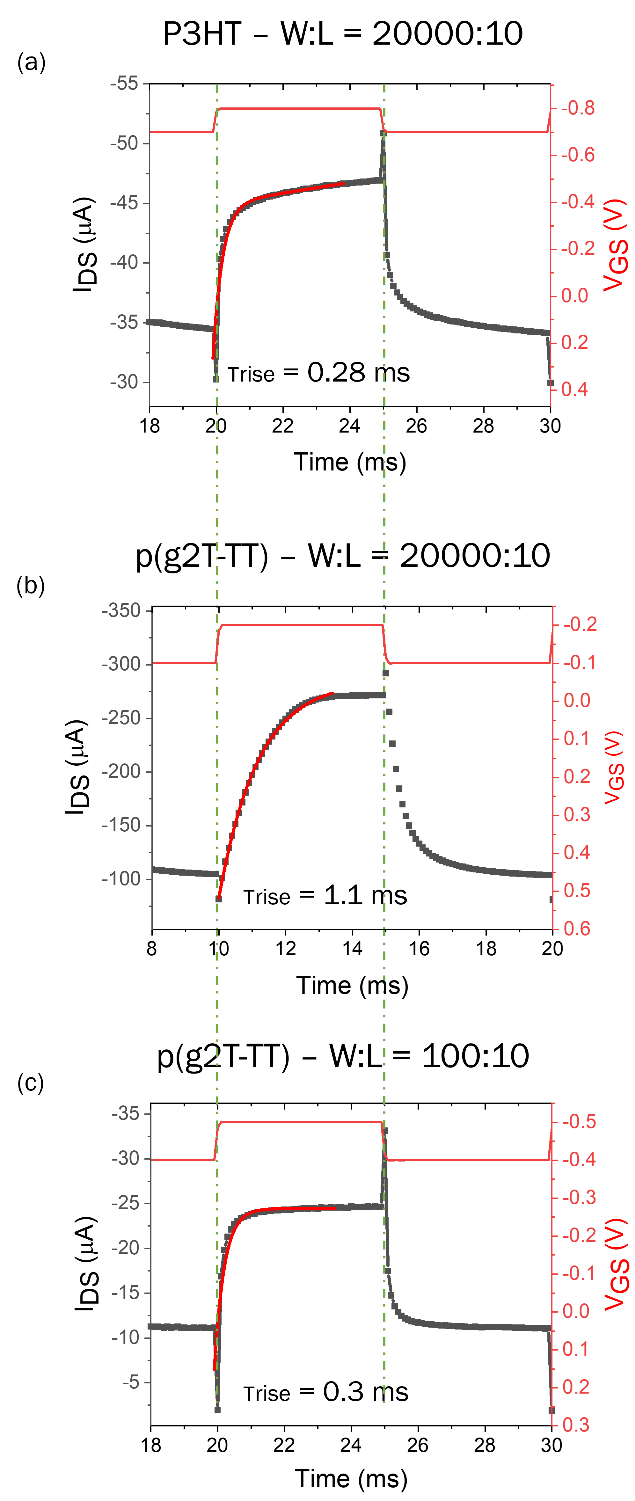
**Figure SI.6:** Time response for (a) P3HT-based EGOFET (*W* ~ 2 cm; *L* = 10 μm), (b) p(g2T-TT)-based OECT (*W* ~ 2 cm; *L*= 10 μm) and (c) p(g2T-TT)-based OECT (*W* = 100 μm; *L* = 10 μm).

**SI.7) *P*(g2T-TT)-based OECTs with “Small Area Transistor” 100:10 W:L**

While the electrical measurements of p(g2T-TT)-based OECTs presented in the main manuscript refer to transistors with a channel width *W* ~ 2 cm and channel length *L* = 10 μm, here we recorded hiPSC-CM biological signals using a substantially smaller transistor geometry, namely *W* = 100 μm and *L* = 10 μm (**Figure SI.7(a)**). The transfer characteristic shows that both current and *g_m_​* scale with device geometry (**Figure SI.7(b)**). Although the smaller geometry results in faster device dynamics compared to the larger p(g2T-TT) transistors, it was nevertheless insufficient to capture the characteristic AP morphology of hiPSC-CMs (**Figure SI.7(c)**).


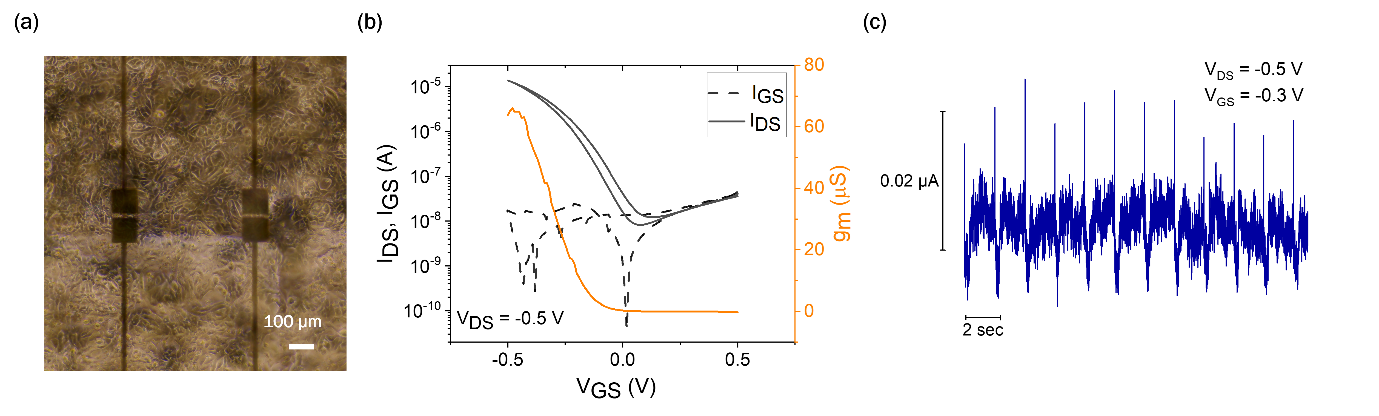


**Figure SI.7**: (a) Optical microscopy of the p(g2T-TT)-based OECTs with W = 100 μm and L = 10 μm in the presence of cells. (b) Representative transfer characteristics and associated extracted transconductance for the devices reported in (a). (c) Recording trace for V_DS_ = ~~-0.3 V~~ -0.5 V and V_GS_ = ~~-0.5 V~~ -0.3 V showing a distorted AP shape with low SNR due to a weak coupling.

**SI. 8) Contact Angle Measurement**

The investigation of the cell-polymer interaction was first evaluated by comparing the surface wettability of the two polymers, by means of contact angle measurements performed using Milli-Q water droplets (5 μL) onto bare polymers. As shown in **Figure SI.8(a)**, right after the droplet deposition P3HT sample shows a mean contact angle of 104.5°, indicating a hydrophobic surface, whereas p(g2T-TT) exhibits a significantly lower value of 71°, consistent with the more hydrophilic nature of the polymer, given by its lateral glycolated chains ^[4]^. These results are summarized in **Figure SI.8(b)** which reports consistent and narrow statistical distribution of the individual data for the two polymers.


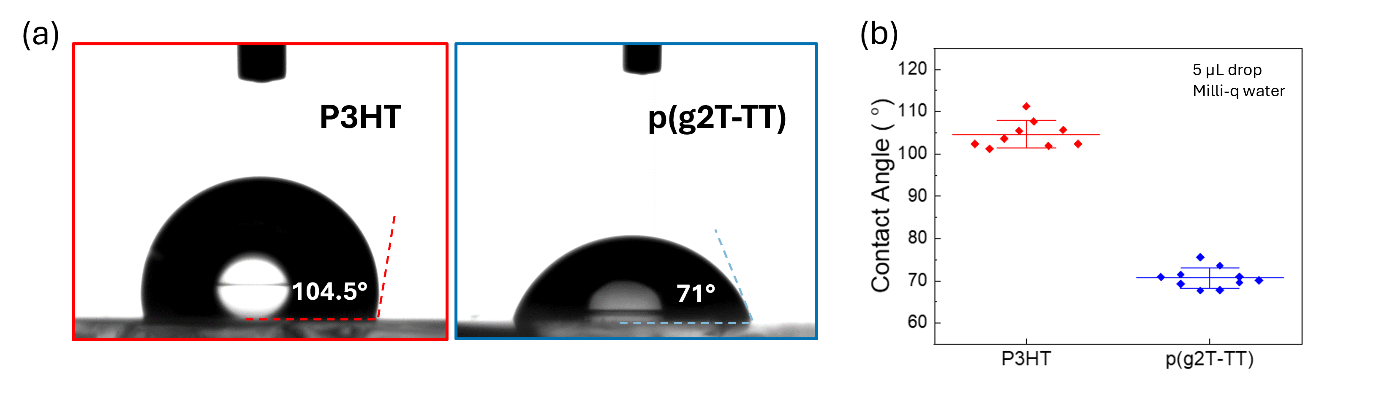
**Figure SI.8**: Contact angle measurements of P3HT and p(g2T-TT) surfaces. (a) Representative images of static water contact angles measured using 5 μL Milli-Q water droplets on inkjet-printed P3HT (left) and p(g2T-TT) (right). Mean values of contact angles for the two polymers are reported; P3HT exhibits a higher contact angle (104.5°), indicating higher hydrophobicity, while p(g2T-TT) shows a lower angle (71°), consistent with enhanced hydrophilicity. (b) Summary plot of contact angle values for multiple measurements on each polymer (N = 9 for P3HT and N = 10 for p(g2T-TT)), confirming the statistically significant difference in wettability.

**SI.9) Atomic Force Microscopy (AFM) Analysis of a Printed p(g2T-TT) Transistor**
To assess the nanoscale morphology of the active layers and evaluate possible correlations between surface topography and cell-substrate coupling, Atomic Force Microscopy (AFM) measurements were carried out on printed p(g2T-TT) OECTs. The analysis provides height maps over different length scales, enabling the extraction of root-mean-square (RMS) roughness values across representative regions of the channel, source/drain electrodes, and interfacial boundaries. This data allow us to quantify film uniformity, identify mesoscale features introduced during the printing process, and determine local thickness variations (**Figure SI.9(g)**). The results reported below highlight the characteristic surface morphology of p(g2T-TT) films, which is subsequently compared to that of P3HT devices in Section SI.10.


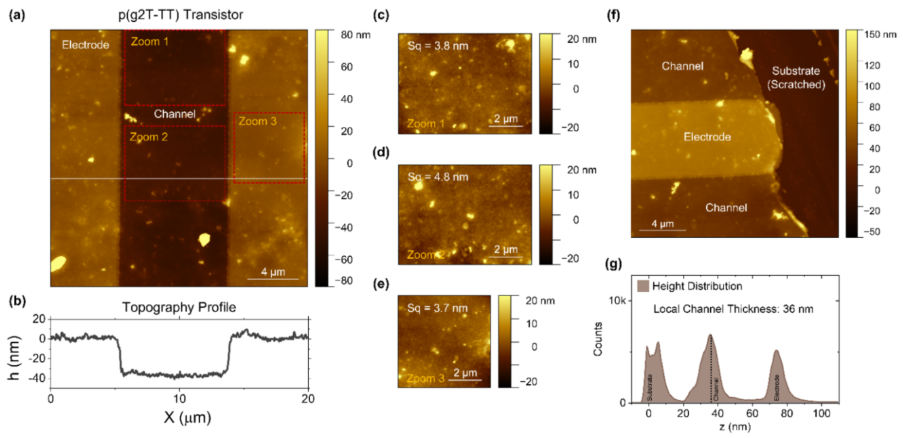
**Figure SI.9**: Atomic force microscopy (AFM) analysis of a printed p(g2T-TT) transistor. (a) Large-area AFM height map (20×20 µm^2^, 512×512 pixels) showing the device architecture, including the source/drain electrode and channel region; red dashed boxes indicate regions selected for roughness analysis (Zoom 1-3), and the white line marks the position of the height profile. (b) Topography line profile extracted along the white line in (a), highlighting the step height between the channel and electrode regions. (c-e) AFM height maps of the p(g2T-TT) surface acquired at the locations marked in (a): (c) Zoom 1 (7.81×5.86 µm^2^, 200×150 pixels), (d) Zoom 2 (7.81×5.86 µm^2^, 200×150 pixels), and (e) Zoom 3 (5.47×5.47 µm^2^, 140×140 pixels), with corresponding local RMS roughness values Sq = 3.8, 4.8, and 3.7 nm, respectively. (f) AFM height map (17.58×17.58 µm^2^, 450×450 pixels) across the electrode-channel-substrate (scratched with a tungsten tip) region used to define reference levels for thickness determination. (g) Height distribution extracted from the region in (f), showing distinct substrate, channel, and electrode populations and yielding a local p(g2T-TT) channel thickness of approximately 36 nm. Scale bars and color bars indicate lateral dimensions and height variations in each panel.

**SI.10) Atomic Force Microscopy (AFM) Analysis of a Printed P3HT Transistor**


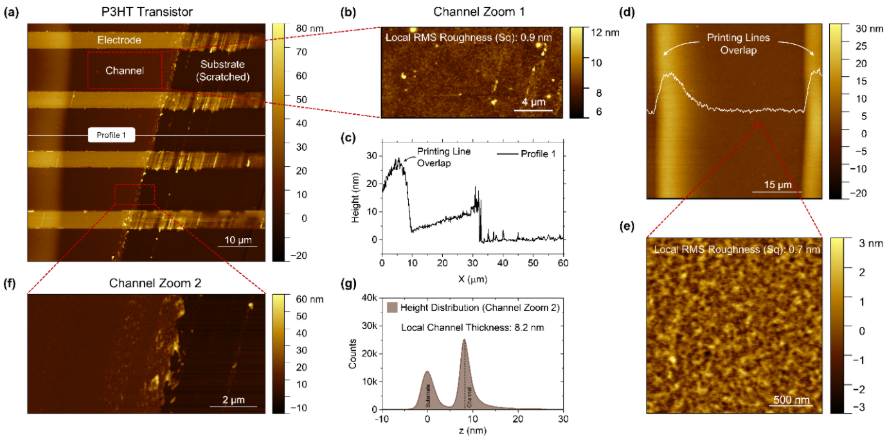

**Figure SI.10**: Atomic force microscopy (AFM) analysis of a printed P3HT transistor. (a) Large-area AFM height map (60×60 µm^2^, 512×512 pixels) showing the device layout, including source/drain electrodes, the P3HT channel, and a scratched substrate reference; the white line indicates the location of the height profile. (b) Zoom of the channel region (Zoom 1, 18.7×9.4 µm^2^, 160×80 pixels), highlighting the local surface morphology, with a local RMS roughness of Sq = 0.9 nm. (c) Height profile (Profile 1) taken across the channel, revealing the step height associated with the P3HT film and overlapping printing lines. (d) AFM height map (52.6×52.6 µm^2^, 280×280 pixels) emphasizing the overlap of adjacent printing lines; the white trace shows the corresponding height profile. (e) High-resolution view of the channel surface (2×2 µm^2^, 256×256 pixels), showing fine-scale morphology with a local RMS roughness of Sq = 0.7 nm. (f) Second channel zoom (Zoom 2, 10×5 µm^2^, 512×256 pixels) near the channel-substrate interface used for median thickness analysis. This region is approximately the central area between the two printing lines, i.e., 15 µm from the edge of a printing line, see (d). (g) Height distribution extracted from Zoom 2, indicating a local P3HT channel median thickness of approximately 8.2 nm. The maximum thickness is approximately 30 nm, as shown in (c). Scale bars and colour bars indicate lateral dimensions and height variations in each panel

**SI.11) Image Processing and Quantification of Vinculin Localization**
To clarify the methodology used to discriminate adhesion-associated vinculin from inactive perinuclear pools, we provide below a schematic overview of the image-processing workflow employed for vinculin quantification. The pipeline was designed to selectively exclude the perinuclear region, where vinculin is predominantly inactive, and to quantify vinculin enrichment close to the cell periphery, where focal adhesions are formed.


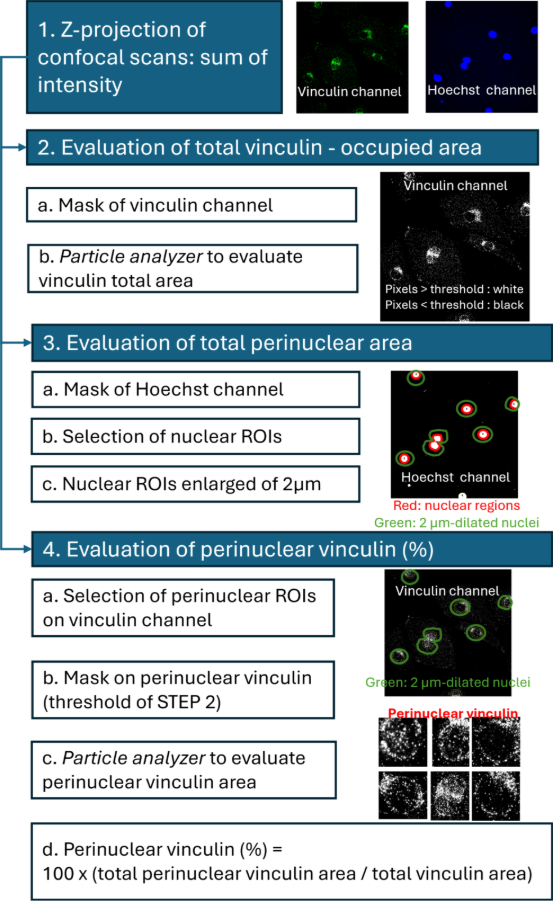
**Figure SI.11:** Flow chart illustrating the processing pipeline used to distinguish and quantify perinuclear and peripheral vinculin. Nuclear regions of interest (ROIs) were extracted from the Hoechst z-projection and expanded by 2 µm to generate perinuclear exclusion ROIs for each nucleus. These enlarged ROIs were combined and overlaid on the vinculin z-projection to define the perinuclear vinculin region. The perinuclear vinculin percentage was calculated as the ratio between the vinculin signal within the enlarged ROIs and the total vinculin-positive area measured across the entire projected vinculin channel. The peripheral vinculin percentage was then obtained by subtracting the perinuclear from the total signal.

**SI.12) Single-Well Device**

A representative photograph of a device with a mounted PLA well is shown in **Figure SI.12**. The well has a cylindrical geometry with a base diameter of 9 mm and a height of 6 mm. The overall dimension of the single-well device is around 2×2.5 cm^2^.


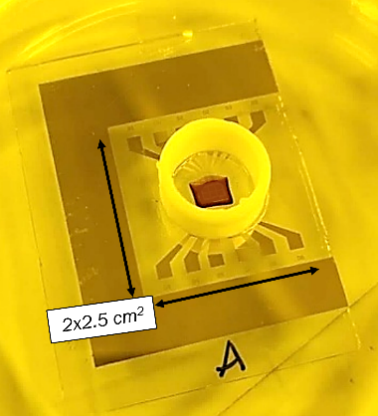


**Figure SI.12:** Representative device architecture bearing the semiconductor on the gold electrodes at the center, with no cells nor cell medium*.*

**SI.13) SU8 Insulation Layer Thickness**

AFM line profiling across a scratched substrate reference revealed an SU8 insulation layer thickness of approximately 5.45 µm (**Figure SI.13**).


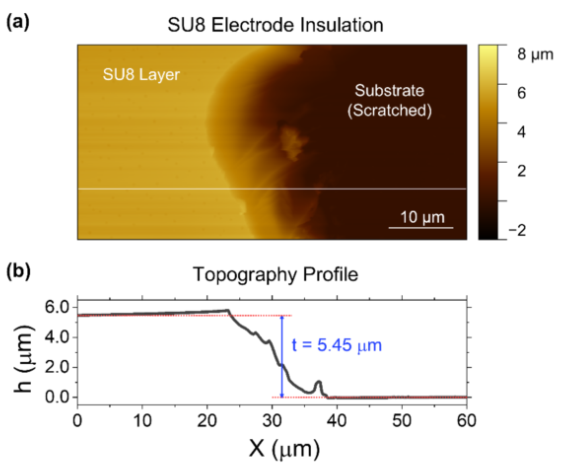


**Figure SI.13:** Atomic force microscopy (AFM) characterization of SU-8 electrode insulation. (a) AFM height map (60×30 µm^2^, 512×256 pixels) showing the SU-8 insulating layer adjacent to a scratched substrate region used as a height reference; the white line indicates the position of the line scan. (b) Topography profile extracted along the white line in (a), revealing a step height corresponding to the SU-8 insulation thickness. The measured SU-8 layer thickness is approximately 5.45 µm. Scale bars and colour bars indicate lateral dimensions and height variations.

[1] A. Kyndiah, G. Z. Zemignani, C. Ronchi, G. Tullii, A. Khudiakov, G. Iachetta, S. Chiodini, R. Moreddu, F. A. Viola, P. J. Schwartz, G. Gomila, F. De Angelis, L. Sala, M. R. Antognazza, M. Caironi, *Nat Commun* 2025, *16*, 8143.

[2] L. Sala, D. Ward-Van Oostwaard, L. G. J. Tertoolen, C. L. Mummery, M. Bellin, *Journal of Visualized Experiments* 2017, *2017*.

[3] H. Wickham, *ggplot2 - Elegant Graphics for Data Analysis*, 2nd ed., Springer Cham 2016.

[4] A. Savva, C. Cendra, A. Giugni, B. Torre, J. Surgailis, D. Ohayon, A. Giovannitti, I. McCulloch, E. Di Fabrizio, A. Salleo, J. Rivnay, S. Inal, *Chemistry of Materials* 2019, *31*, 927.
